# Effectiveness of Beta-Blockers in Reducing Mortality and Recurrence After Myocardial Infarction: A Systematic Review of Contemporary and Foundational Evidence

**DOI:** 10.7759/cureus.91371

**Published:** 2025-08-31

**Authors:** Alaa M Popraya, Muhammad Ibrahim, Nouman Anthony, Sidra Anmol, Faizan Ahmed, Bilawal Kamran

**Affiliations:** 1 Acute Medicine, Royal Derby Hospital, Derby, GBR; 2 Internal Medicine, Shalamar Institute of Health Sciences, Lahore, PAK; 3 Internal Medicine, Rehman Medical Institute, Peshawar, PAK; 4 Internal Medicine, Government Samanabad Hospital, Lahore, PAK; 5 General Surgery, Rawalpindi Medical University, Rawalpindi, PAK; 6 Internal Medicine, Nishtar Medical University, Multan, PAK

**Keywords:** beta-blockers, cardiovascular outcomes, mortality, myocardial infarction, reinfarction, secondary prevention, systematic review

## Abstract

Beta-blockers have traditionally been a mainstay in the management of patients recovering from myocardial infarction (MI). However, their role in the era of modern cardiac interventions remains a topic of active discussion. This systematic review evaluated evidence from 1983 to 2014, drawn from PubMed, Embase, Scopus, and CENTRAL, on the effectiveness of beta-blockers in reducing mortality and recurrent cardiovascular events in adults following MI. After a comprehensive screening process, four eligible studies (two randomized controlled trials, one post hoc analysis, and one observational study) were included, encompassing a total of 19,078 participants with varied clinical settings and patient profiles. Across these studies, beta-blocker therapy was consistently associated with reductions in all-cause mortality, cardiovascular mortality, and recurrent MI, particularly among individuals with reduced left ventricular function. Some benefit was also observed in those with preserved function, though with less consistency. The magnitude of benefit included a 23-26% reduction in overall mortality and up to a 41% reduction in recurrent MI. Despite differences in study design, patient characteristics, and treatment protocols, the overall findings support the continued use of beta-blockers in post-infarction care. Limitations included heterogeneity in populations, beta-blocker regimens, and study eras, highlighting the need for individualized treatment approaches. Nonetheless, these results align with current guidelines from the American College of Cardiology/American Heart Association and the European Society of Cardiology, reaffirming the relevance of beta-blockers in improving outcomes in this patient population.

## Introduction and background

Myocardial infarction (MI), commonly referred to as a heart attack, remains one of the leading causes of morbidity and mortality worldwide, accounting for an estimated 9 million deaths annually, according to recent global data [1]. A heightened risk of sudden cardiac death, recurrent ischemic events, and progressive heart failure characterizes the early period following an acute MI. As a result, optimizing secondary prevention strategies in this critical phase is essential to improving long-term outcomes [2]. Beta-adrenergic blockers, or beta-blockers, have been a cornerstone of post-MI management for several decades due to their capacity to reduce myocardial oxygen demand by lowering heart rate and contractility, prevent arrhythmias through suppression of adrenergic activity, and attenuate adverse ventricular remodeling [3].

The use of beta-blockers in the post-MI setting initially gained prominence following landmark randomized controlled trials (RCTs) conducted in the 1980s and 1990s, such as the Beta-Blocker Heart Attack Trial (BHAT) [4], which showed a 26% reduction in total mortality, and the CAPRICORN study [5], which reported a 23% relative reduction in all-cause mortality and a 41% reduction in recurrent MI with carvedilol therapy. These trials demonstrated a significant reduction in sudden cardiac death and long-term mortality compared to placebo. However, with the advent of contemporary revascularization techniques like primary percutaneous coronary intervention (PCI), the widespread use of dual antiplatelet therapy (DAPT), statins, and angiotensin-converting enzyme (ACE) inhibitors, questions have emerged regarding the continued additive benefit of beta-blockers in modern clinical practice, particularly in patients with preserved left ventricular ejection fraction (LVEF ≥50%) [6]. In contrast, patients with a reduced LVEF (<40%) remain at a higher risk and may derive a greater benefit. These advances in therapy may attenuate or overlap with the mechanisms by which beta-blockers exert their protective effects, necessitating a reassessment of their role in today’s treatment paradigm.

Recent studies published between 2010 and 2015, including large-scale observational analyses and post hoc evaluations from major trials such as CHARISMA [7], have provided updated insights into the role of beta-blockers in diverse patient subgroups. While some evidence supports their ongoing benefit in reducing cardiovascular mortality and recurrent MI, especially in high-risk patients or those with reduced LVEF, other data suggest a diminished or uncertain effect in lower-risk cohorts or those with preserved cardiac function. Importantly, heterogeneity in the beta-blocker class (e.g., cardioselective vs. non-selective agents with intrinsic sympathomimetic activity) and dosing regimens is clinically relevant, as these pharmacological differences may influence both efficacy and tolerability. Potential safety concerns, such as bradycardia, hypotension, fatigue, and worsening heart failure in susceptible individuals, also warrant consideration in tailoring therapy. These conflicting results underscore the need for a systematic synthesis of current evidence to clarify the therapeutic impact of beta-blockers in the post-MI population, particularly in the context of modern guideline-directed medical therapy.

Therefore, the objective of this study is to systematically evaluate the effectiveness of beta-blockers in reducing mortality and recurrent cardiovascular events in patients who have experienced a MI. To ensure a structured and focused approach, we have utilized the population, intervention, comparison, and outcome (PICO) framework [8], which is widely recognized in systematic reviews as a robust method to define eligibility criteria, guide literature searches, and enhance the reproducibility and transparency of the study selection process.

## Review

Materials and methods

Search Strategy

A systematic literature search was conducted in accordance with the Preferred Reporting Items for Systematic Reviews and Meta-Analyses (PRISMA) guidelines [9] to ensure methodological transparency and reproducibility. The search included the following databases: PubMed, Embase, Scopus, and the Cochrane Central Register of Controlled Trials (CENTRAL). Studies published from database inception through June 30, 2024 (last search date), were considered. The search strategy employed a combination of Medical Subject Headings (MeSH) and free-text terms, including “beta-blockers”, “myocardial infarction”, “mortality”, “reinfarction”, “post-MI”, and “secondary prevention”. Boolean operators (AND, OR) were used to combine search terms logically. References of retrieved articles and prior reviews were manually screened to identify additional eligible studies. All records were compiled using reference management software, and duplicates were removed before screening.

Eligibility Criteria

The inclusion criteria for this review were developed based on the PICO model. The population included adult patients (≥18 years) with a confirmed diagnosis of MI, including ST-elevation MI (STEMI) and non-ST-elevation MI (NSTEMI). The intervention was beta-blocker therapy initiated after MI, irrespective of the agent, dose, or duration. The comparison group consisted of patients who either received a placebo, no beta-blocker therapy, or standard care without beta-blockade. The outcomes of interest were all-cause mortality, cardiovascular mortality, and recurrent MI (nonfatal reinfarction).

Studies were included if they (1) were RCTs or high-quality observational studies, (2) involved human subjects diagnosed with MI, (3) compared outcomes in beta-blocker users versus non-users or placebo groups, and (4) reported at least one of the primary outcomes of interest. Only studies published in English were considered, as translation resources were unavailable; we acknowledge that this restriction may introduce a potential language bias. Exclusion criteria included studies focusing exclusively on unstable angina, reviews, meta-analyses, editorials, conference abstracts, animal studies, and trials where beta-blocker use was not clearly defined or not assessed as an independent intervention. Studies lacking sufficient statistical data or relevant outcome reporting for our PICO were also excluded.

Study Selection and Data Extraction

Titles and abstracts were screened independently by two reviewers, followed by a full-text assessment of potentially eligible articles. A PRISMA flow diagram was constructed to illustrate the selection process. Data from each included study were extracted independently by the same two reviewers using a standardized data collection form. Extracted variables included study design, year of publication, sample size, patient characteristics (e.g., type of MI, LVEF), type and dosage of beta-blocker used, comparison group details, follow-up duration, and reported outcomes (mortality and recurrent MI). Where necessary, hazard ratios, relative risk reductions, absolute event rates, and confidence intervals were recorded. Disagreements during the extraction process were resolved by consensus or consultation with a third reviewer.

Data Analysis and Synthesis

Due to heterogeneity in study designs, patient populations, and outcome definitions, a qualitative narrative synthesis was conducted. Both RCTs and observational studies were included to provide a comprehensive assessment across different levels of evidence; differences in methodological rigor were addressed by formally assessing risk of bias with design-specific tools. Studies were grouped and analyzed based on methodological design (RCT vs. observational), LVEF categorization (reduced vs. preserved), and outcome type. Results were summarized in structured tables to allow direct comparison of effect sizes and risk reductions across studies. Heterogeneity was explored qualitatively, and statistical pooling was considered but not feasible due to the small number of studies and inconsistent outcome reporting. Risk of bias was assessed using the Cochrane Risk of Bias 2.0 tool (Cochrane Collaboration, London, UK) [10] for RCTs and the ROBINS-I tool for observational studies [11]. Disagreements in risk of bias assessment were resolved by discussion until consensus was reached. To minimize publication bias, the reference lists of eligible studies were screened; however, study protocols and trial registries were not systematically searched. Findings were interpreted in light of study quality and clinical relevance, with attention given to consistency and applicability across modern post-MI care settings.

Results

Study Selection Process

**Figure 1:**
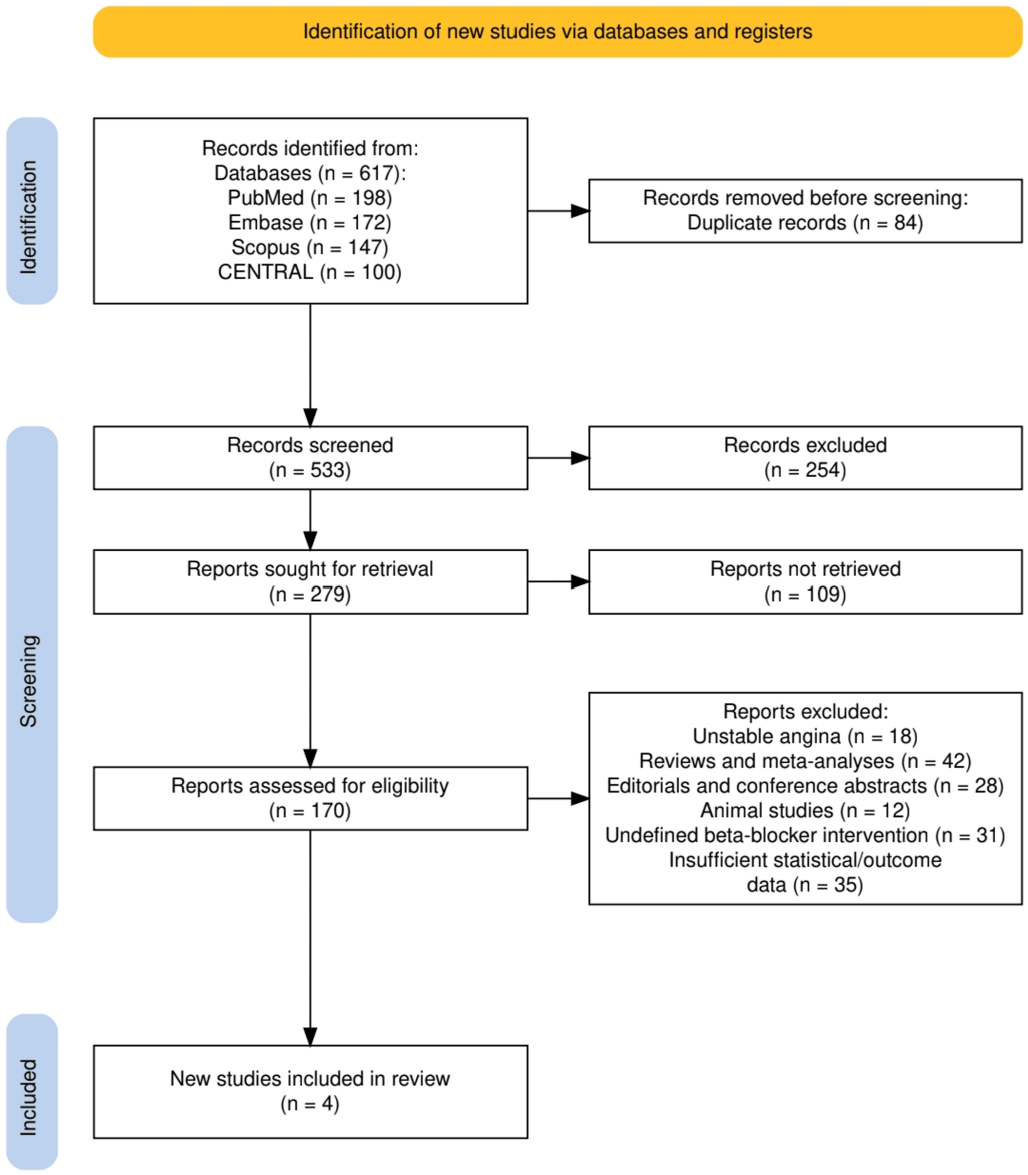
PRISMA flowchart representing the study selection process PRISMA: Preferred Reporting Items for Systematic reviews and Meta-Analyses

The study selection process adhered to the PRISMA guidelines and is illustrated in Figure 1. A total of 617 records were identified through four electronic databases: PubMed (n = 198), Embase (n = 172), Scopus (n = 147), and CENTRAL (n = 100). After removing 84 duplicate records, 533 studies were screened based on titles and abstracts. Of these, 254 were excluded because they did not meet the inclusion criteria. Full texts of 279 articles were sought for retrieval, but 109 could not be accessed, leaving 170 studies for full-text eligibility assessment. Following detailed evaluation, 166 studies were excluded based on specific criteria, including focus on unstable angina (n = 18), review or meta-analysis articles (n = 42), editorials and conference abstracts (n = 28), animal studies (n = 12), undefined beta-blocker intervention (n = 31), and insufficient statistical or outcome data (n = 35). Ultimately, four studies met the inclusion criteria and were included in the final review.

Characteristics of the Selected Studies

The characteristics of the four studies included in this systematic review are summarized in Table 1. These studies represent a combination of RCTs and a high-quality post hoc analysis, each investigating the effectiveness of beta-blocker therapy in reducing adverse outcomes following MI. Sample sizes ranged from 1,959 to 8,510 participants, with variability in MI type, LVEF, and clinical settings. One study focused on STEMI patients undergoing primary PCI, including those with preserved LVEF, and found a significant reduction in all-cause mortality among those discharged on beta-blockers (2.1% vs. 3.6%; HR 0.46). Another trial targeting patients with LVEF <40% reported a 23% relative reduction in total mortality and a 41% reduction in recurrent MI with carvedilol therapy over 15 months. A third study, based on a post hoc analysis of a large trial population, demonstrated a 31% reduction in composite cardiovascular outcomes and a 38% reduction in recurrent MI among beta-blocker users with a history of MI, despite no significant difference in overall mortality. The oldest trial included showed that long-term propranolol use after MI resulted in a 26% decrease in total mortality, along with notable reductions in cardiovascular death, sudden death, and reinfarction during a 25-month follow-up. Despite differences in trial designs, population characteristics, and timeframes, all studies consistently reported beneficial effects of beta-blockers in the post-MI setting.

**Table 1:** Summary of all of the included studies in the review RCT: randomized controlled trial, CAPRICORN: Carvedilol Post-Infarct Survival Control in Left Ventricular Dysfunction, CHARISMA: Clopidogrel for High Atherothrombotic Risk and Ischemic Stabilization, Management, and Avoidance, BHAT: Beta-Blocker Heart Attack Trial, MI: myocardial infarction, STEMI: ST-elevation myocardial infarction, LVEF: left ventricular ejection fraction, EF: ejection fraction, HF: heart failure, BB: beta-blocker, ACEI: angiotensin-converting enzyme inhibitor, CV: cardiovascular, RRR: relative risk reduction, HR: hazard ratio, IQR: interquartile range

Study (author, year)	Study design	Population (sample size, MI type, LVEF)	Intervention (beta-blocker type, dose)	Comparison group	Primary outcomes	Follow-up duration	Key findings
Yang et al., 2014 [12]	RCT (propensity-score matched analysis)	8,510 STEMI patients undergoing PCI; subgroup analysis includes EF >40%	Beta-blocker at discharge; specific agents/doses not specified	No beta-blocker at discharge	All-cause mortality	Median 367 days (IQR: 157–440 days)	BB group had lower all-cause mortality (2.1% vs. 3.6%, p<0.001); HR 0.46 after matching
Otterstad and Ford, 2002 [13]	RCT (CAPRICORN)	1,959 post-MI patients with a mean LVEF 33% (all <40%)	Carvedilol; titrated after ≥48h of ACEI initiation	Placebo + standard post-MI therapy	Total mortality and recurrent MI	Mean 15 months	Mortality reduced from 15.3% to 11.9% (RRR 23%); recurrent MI reduced from 5.8% to 2.3% (RRR 41%)
Bangalore et al., 2014 [7]	Post hoc analysis of an RCT (CHARISMA trial)	4,772 patients with prior MI (subset of larger cohort); no HF; LVEF not reported	Beta-blocker use at baseline; type/dose not specified	No beta-blocker at baseline	Composite: nonfatal MI, stroke, CV mortality	Median 28 months	In prior MI group: BB reduced composite events (HR 0.69, p=0.021); lower recurrent MI (HR 0.62)
Goldstein, 1983 [14]	RCT (BHAT)	3,837 post-MI patients (men and women); LVEF not specified	Propranolol; dose not specified; initiated 5–21 days post-MI	Placebo	Total mortality, CV mortality, sudden death, and reinfarction	Mean 25 months	Propranolol reduced total mortality by 26%, CV mortality by 26%, sudden death by 28%, and reinfarction by 23%

Quality Assessment

The quality assessment of the included studies is presented in Table 2, with appropriate appraisal tools applied according to study design to evaluate methodological rigor and risk of bias. One observational study, assessed using the ROBINS-I tool, demonstrated a moderate risk of bias due to its non-randomized nature, despite using propensity score matching to reduce confounding and having a large, well-balanced sample. Among the RCTs evaluated using the Cochrane Risk of Bias 2.0 tool, one was identified as having a low risk of bias, supported by robust methodology, clear endpoints, and minimal attrition, indicating high internal validity. A post hoc analysis of a randomized trial, also assessed using the ROBINS-I tool, revealed a moderate to serious risk of bias, primarily due to its retrospective design, lack of random beta-blocker assignment, and reliance on subgroup data. Another early randomized trial was rated as low risk of bias, with well-defined outcomes, comprehensive follow-up, and rigorous blinding procedures, despite being conducted in an earlier treatment era. Collectively, the included evidence comprises two high-quality randomized trials and two well-executed observational studies, forming a solid basis for drawing clinically meaningful conclusions.

**Table 2:** Quality assessment of the included studies in the review CAPRICORN: Carvedilol Post-Infarct Survival Control in Left Ventricular Dysfunction, CHARISMA: Clopidogrel for High Atherothrombotic Risk and Ischemic Stabilization, Management, and Avoidance, BHAT: Beta-Blocker Heart Attack Trial, RCT: randomized controlled trial, BB: beta-blocker

Study (author, year)	Appraisal tool used	Tool criteria applied	Quality assessment summary	Overall judgment
Yang et al., 2014 [12]	ROBINS-I (for observational studies with intervention)	Confounding, participant selection, classification of intervention, deviations from intended interventions, missing data, measurement of outcomes, selection of reported results	Propensity-matched design reduces confounding; large sample size; real-world relevance; baseline characteristics well balanced; residual confounding likely but minimized	Moderate risk of bias
Otterstad and Ford, 2002 (CAPRICORN) [13]	Cochrane Risk of Bias 2.0 (RCTs)	Randomization process, deviations from intended interventions, missing outcome data, measurement of outcomes, and selection of the reported result	Double-blind RCT with placebo control; adequate randomization; outcome definitions clear; follow-up robust; low attrition	Low risk of bias
Bangalore et al., 2014 (CHARISMA post hoc) [7]	ROBINS-I (post hoc observational analysis)	Confounding, selection of participants, intervention classification, deviations from intended interventions, missing data, and outcome measurement	Well-conducted propensity score analysis; no random assignment to BBs; limited control over baseline BB usage; subgroup and post hoc analysis inherently weaker	Moderate risk of bias
Goldstein, 1983 (BHAT) [14]	Cochrane Risk of Bias 2.0 (RCTs)	Randomization, blinding, outcome measurement, follow-up, reporting	Classic double-blind placebo-controlled RCT; clearly defined outcomes; strong internal validity; older era, but methodology was rigorous for its time	Low risk of bias

Discussion

The findings of this systematic review demonstrate a consistent benefit of beta-blocker therapy in reducing both mortality and recurrent MI in post-MI patients, with varying degrees of effect depending on patient characteristics and study design. Across the four selected studies, beta-blockers were associated with statistically significant reductions in all-cause mortality, cardiovascular mortality, and the incidence of recurrent MI. In the large, propensity-matched cohort study by Yang et al. [12], involving 8,510 STEMI patients who underwent primary PCI, all-cause mortality was significantly lower in the beta-blocker group (2.1%) compared to those not discharged on beta-blockers (3.6%), with a hazard ratio (HR) of 0.46 (95% CI: 0.27-0.78, p = 0.004). The CAPRICORN trial by Otterstad and Ford [13], a double-blind RCT enrolling 1,959 post-MI patients with a mean LVEF of 33%, showed that carvedilol reduced total mortality from 15.3% to 11.9%, yielding a relative risk reduction (RRR) of 23% and an absolute risk reduction (ARR) of 3.4%. Additionally, the incidence of recurrent MI was reduced from 5.8% to 2.3% (RRR 41%, ARR 2.3%), with a number needed to treat of 28 to prevent one death over the full follow-up period. In the post hoc analysis from the CHARISMA trial [7], Bangalore et al. analyzed 4,772 patients with a history of MI and no heart failure, finding that beta-blocker use was associated with a 31% lower risk of the composite outcome of nonfatal MI, stroke, or cardiovascular death (7.1% vs. 10.2%; HR: 0.69; 95% CI: 0.50-0.94; p = 0.021), and a 38% lower risk of recurrent MI (3.4% vs. 4.9%; HR: 0.62; 95% CI: 0.39-1.00; p = 0.049). Lastly, the landmark BHAT trial [14] demonstrated that propranolol therapy significantly reduced total mortality by 26%, cardiovascular mortality by 26%, sudden cardiac death by 28%, and coronary incidence (reinfarction plus CHD mortality) by 23% over a mean follow-up of 25 months in 3,837 post-MI patients.

The findings of this review are largely consistent with prior evidence and reinforce the recommendations of leading cardiology guidelines such as those by the American College of Cardiology/American Heart Association (ACC/AHA) [15] and the European Society of Cardiology (ESC) [16], which advocate for long-term beta-blocker therapy in all patients following MI, especially those with reduced ejection fraction. Our results align with previous meta-analyses that have shown beta-blockers to significantly lower all-cause mortality and recurrent ischemic events in post-MI patients [17,18]. However, unlike earlier studies conducted before the widespread use of modern reperfusion therapies, statins, and ACE inhibitors, this review incorporates more contemporary data that reflects the current treatment landscape [19]. The consistency of benefit in studies such as Yang et al. [12] and CAPRICORN [13], even in the context of primary PCI and optimized secondary prevention, suggests that beta-blockers retain clinical value despite advances in MI care. Nonetheless, our review also highlights emerging questions around the magnitude of benefit in certain low-risk populations, indicating that universal prescription without risk stratification may require further scrutiny.

The clinical implications of our findings suggest that beta-blockers remain a critical component of post-MI pharmacologic therapy, particularly in patients with a reduced LVEF, who derive clear benefits in terms of mortality and reinfarction [20]. In contrast, the evidence for patients with preserved LVEF is less definitive but still supports the role of beta-blockers in reducing recurrent events, as demonstrated in studies like Yang et al. [12] and Bangalore et al. [7]. From a clinical decision-making perspective, our review reinforces the use of guideline-concordant therapy in high-risk patients, while also suggesting the potential for more individualized strategies in lower-risk groups [21]. Clinicians should weigh factors such as LVEF, comorbidities, and hemodynamic status when prescribing beta-blockers, rather than employing a one-size-fits-all approach [22]. The data support continued adherence to established guidelines but also call for nuanced application in real-world settings to optimize outcomes and minimize overtreatment.

The studies included in this review exhibited some degree of heterogeneity that may account for variations in outcomes. Differences in study design, particularly the inclusion of both RCTs (e.g., CAPRICORN, BHAT) and observational or post hoc analyses (e.g., CHARISMA, Yang et al.), affect the internal validity and generalizability of findings. Additionally, the types and doses of beta-blockers were not consistently reported or standardized, which may have influenced efficacy across trials. Timing of initiation varied as well, with some studies initiating therapy days after MI, while others assessed beta-blocker use at discharge. Patient populations also differed significantly: some trials included only those with reduced LVEF, while others included broader groups with preserved function or no heart failure. These variables introduce clinical and methodological heterogeneity that must be considered when interpreting pooled trends and applying them to individual patients.

One of the major strengths of this review is its methodological rigor. The literature search was conducted systematically in compliance with the PRISMA guidelines, ensuring transparency and reproducibility. We included both classic RCTs and recent real-world data, allowing for a comprehensive synthesis that bridges the historical and contemporary eras of cardiovascular care. By integrating landmark trials, such as BHAT [14] and CAPRICORN [13], with newer evidence from registry-based studies and post hoc analyses, this review provides a comprehensive perspective on beta-blocker efficacy. The inclusion of studies across a spectrum of risk profiles, treatment settings, and LVEF categories enhances the external validity of our conclusions and underscores their relevance to diverse patient populations.

Despite these strengths, the review is subject to several limitations. There was considerable variability in the types, doses, and treatment durations of beta-blockers reported across studies, which may have influenced the consistency of the outcomes. Some studies, such as CHARISMA [7], were post hoc analyses and inherently less robust than prospectively designed trials. Furthermore, adherence data were not consistently reported, which limited our ability to assess the true duration and intensity of beta-blocker exposure accurately. Several of the included studies, particularly BHAT [14], were conducted in the pre-PCI era, which may limit the applicability of their findings to today’s treatment context. Additionally, the potential for publication bias cannot be ruled out, particularly in the absence of access to unpublished negative studies that may have influenced the balance of available evidence.

Future research should focus on addressing current gaps by conducting RCTs specifically targeting patients with preserved LVEF following MI, where the benefit of beta-blockers remains uncertain [23]. Long-term observational studies examining the outcomes of beta-blocker discontinuation in stable post-MI patients are also warranted to inform deprescribing decisions. Comparative trials examining different beta-blocker classes (e.g., cardioselective vs. non-selective) and dosing regimens could further refine clinical practice. Moreover, there is a need for studies in underrepresented populations such as women, the elderly, and patients from low- and middle-income countries, who may exhibit different risk profiles and treatment responses. Personalized approaches to beta-blocker therapy that incorporate genomic, demographic, and comorbidity data could ultimately enhance patient outcomes and optimize therapeutic strategies.

## Conclusions

This systematic review reaffirms the clinical benefit of beta-blocker therapy in reducing mortality and recurrent ischemic events following MI, particularly in patients with reduced ejection fraction. The evidence remains supportive of current guideline recommendations, although the data suggest that benefits may vary by patient subgroup and risk profile. By synthesizing findings from both historical and contemporary studies, our review contributes to a more nuanced understanding of beta-blocker utility in the post-MI setting. It underscores the need for personalized, evidence-based treatment strategies going forward.
